# Evolutionary game theory of continuous traits from a causal perspective

**DOI:** 10.1098/rstb.2021.0507

**Published:** 2023-05-08

**Authors:** Jussi Lehtonen, Jun Otsuka

**Affiliations:** ^1^ Department of Biological and Environmental Science, University of Jyväskylä, 40014 Jyväskylä, Finland; ^2^ Department of Philosophy, Kyoto University, Yoshida-Hommachi, 606-8501 Kyoto, Japan

**Keywords:** causality, causal graph, causal derivative, path coefficient, game theory, evolution

## Abstract

Modern evolutionary game theory typically deals with the evolution of continuous, quantitative traits under weak selection, allowing the incorporation of rich biological detail and complicated nonlinear interactions. While these models are commonly used to find candidates for evolutionary endpoints and to approximate evolutionary trajectories, a less appreciated property is their potential to expose and clarify the causal structure of evolutionary processes. The mathematical step of differentiation breaks a nonlinear model into additive components which are more intuitive to interpret, and when combined with a proper causal hypothesis, partial derivatives in such models have a causal meaning. Such an approach has been used in the causal analysis of game-theoretical models in an informal manner. Here we formalize this approach by linking evolutionary game theory to concepts developed in causal modelling over the past century, from path coefficients to the recently proposed causal derivative. There is a direct correspondence between the causal derivative and the derivative used in evolutionary game theory. Some game theoretical models (e.g. kin selection) consist of multiple causal derivatives. Components of these derivatives correspond to components of the causal derivative, to path coefficients, and to edges on a causal graph, formally linking evolutionary game theory to causal modelling.

This article is part of the theme issue ‘Half a century of evolutionary games: a synthesis of theory, application and future directions’.

## 1. Introduction

Game theory and its extensions have become a central part of the evolutionary theorist's toolkit over the last decades, having been explicitly defined in the work of Maynard Smith & Price [1–3], but with precursors in earlier work (particularly in sex ratio theory [4,5]). Originating in economics [6] and later imported into evolutionary biology, the central idea of evolutionary game theory (EGT) is to model situations where the fitness consequences of a given behaviour (or other trait) depend on what other individuals in the population are doing. EGT has been very influential in our understanding of many central questions in evolutionary biology, including sex ratio theory [7], the evolutionary origin of the two sexes [3,8] and its consequences [9,10] and many others (see [11] for more examples).

EGT was first introduced in a pay-off matrix form with discrete strategies [1–3], and while this form of EGT is still relevant in modern literature (e.g. [12]), the form of EGT applied to contemporary biological problems is commonly concerned with continuous traits where individuals may take on any trait value from some continuous range instead of a fixed set of discrete values (lack of appreciation for this distinction has played a role in some debates regarding evolutionary theory, as noted by [13]). Aspects of this continuous trait form of EGT were already present in Hamilton's [5] work on sex ratios, and it was more precisely defined in Maynard Smith's classic treatment ([3] their appendix H) in a simple static form. It has since been merged with several other aspects of evolutionary theory, such as models where one explicitly considers the evolutionary dynamic as a sequence of successive allele replacements (going by a variety of names e.g. adaptive dynamics, invasion analysis, or trait substitution sequence models: [14–18]), quantitative genetics [19–21] and kin selection [17,22–24]. While EGT was initially focused on finding stable endpoints [1,3] for trait evolution, these subsequent extensions bring a dynamic aspect to evolutionary game theory, characterize equilibria (as well as limitations of equilibrium concepts: [25,26]) in richer detail, and connect game theory to social evolution theory. In this article we take these as organic aspects of modern evolutionary game theory, focusing on EGT as it is applied to continuous quantitative traits.

EGT has a dimension which is often left implicit: causal analysis of fitness and natural selection in a potentially very complicated theoretical model. On a more general level outside of game theory, the causal structure of evolutionary theory has been a major topic of research interest in recent years (e.g. [27–32]). Much of this research has focused on additive causal effects with no frequency dependence, while questions in adaptive evolution commonly hinge on non-additive, frequency-dependent effects, and frequency dependence is indeed central to EGT: in the absence of frequency-dependence, a continuous EGT model effectively reduces to a standard optimization model (‘simple optimization’ in the language of [33]). It has been argued that weak selection models and associated (partial) derivatives can be a powerful aid for fine-grained causal analysis of natural selection that can supplement causal interpretation using other methods and can incorporate non-additive causal effects [34]. At the same time and in a different context, Henshaw *et al*. [35] independently introduced the concept of the ‘causal derivative’. In this article, we combine these views, examine the relationship between EGT and the theory of causal modelling, and integrate EGT in its continuous form with the causal derivative of Henshaw *et al*. [35]. This unites the causal analysis of EGT with the framework of tools used in causal modelling and permits the use of a common language and common set of concepts that is used in the causal analysis of theory as well as that of empirical data [36].

From a practical perspective, this is not entirely new: for example, the sex ratio model of Taylor [37] from more than four decades ago presents an informal causal analysis of partial derivatives arising in a game-theoretical model. We will discuss this model in more detail later. In a paper on kin selection, Frank [38] writes that one can study partial derivatives to learn how biological assumptions translate into effects on fitness (‘costs’ and ‘benefits’ in a kin selection context, but the same idea applies to other models we discuss below) and that these effects can be ‘impossible to obtain intuitively, or by inspecting the mathematical expression for fitness'. Indeed, the process of differentiation seems to ‘extract’ further insight from a model that ultimately the researcher has set up themselves, which may seem counterintuitive given that differentiation removes rather than adds information [34]. A key reason why this works is that differentiation resolves a potentially complicated, nonlinear, frequency-dependent expression for fitness into additive components, and such additive structure is intuitively easier to understand and interpret.

Furthermore, as we see in the next section, under common assumptions of EGT these additive components of fitness appear in the same form in the expression for evolutionary change, so that components of the additive approximation for fitness simultaneously correspond to the proportion of evolutionary change they cause (assuming the absence of confounding in the causal model). Thus, differentiation-based game theory methodology is intimately connected to the recent ‘causal derivative’ introduced in the literature of causal modelling for analysing nonlinear interactions. Most previous causal analyses of EGT models have been relatively informal, often without an explicit link to causal modelling theory—by necessity, because some of the relevant concepts have only been defined recently. We aim to integrate such interpretations with the theoretical framework that currently exists for causal analysis, thus formally linking game-theoretical models and their components to concepts that have been developed over the past century, such as Wright's [39,40] ‘path coefficients’ and the closely related ‘causal derivative’ [35]. Recently, there has been much progress and interest in causal modelling both in terms of general theory [36], and in its application to biological issues (e.g. [30,31]) We hope that by linking evolutionary game theory to some of these developments, evolutionary game theory can be made more accessible to researchers familiar with causal thinking, and similarly, causal modelling can be made more accessible to researchers familiar with evolutionary game theory.

We begin by presenting relevant concepts from EGT and causal modelling. We next examine examples of game-theoretical models, placing them in a formal causal modelling context. We will then discuss extensions of EGT to structured populations (kin selection) and to trans-generational effects (niche construction). Our examples all serve as examples of different applications of causal modelling in EGT. Finally, we will discuss confounding, the difference between the causal derivative and that used in game-theoretical models, and the relation of the ‘phenotypic gambit’ to these topics.

## 2. Unpacking fitness and the selection differential

As we will see, causal models and causal graphs typically model causal influences on *fitness*: roughly speaking, they attempt to tease apart the causal influences of focal traits on the number of offspring of a focal individual. While the concept of fitness is central to evolutionary theory, when we aim to understand adaptation it is equally important to understand selection and the evolutionary *response to selection*. Fitness and selection are of course linked: variation in fitness is necessary for selection to occur.

In this section, we show how fitness relates to the response to selection under characteristic assumptions of contemporary continuous evolutionary game theory. In such a model we begin with a function representing the fitness (or expected fitness) of a focal individual expressing a mutant trait value *x* in a population with mean trait value x*:

2.1 $$w(x,x^{*}).$$

In EGT models x* is often called the ‘resident’ trait value, stemming from a population genetic idea of a rare mutant individual with trait value *x* introduced into a large population with a resident allele coding for trait value x*. Here we will use these terms interchangeably: mean trait value and resident trait value when describing x*. When fitness of a focal individual depends on the traits of other individuals in the population, we say that selection is frequency dependent [11].

The fitness function is therefore a function of two variables, *x* and x*. In evolutionary models we are commonly interested in the evolutionary response (change in the population mean phenotype x*) resulting from such a fitness function, given certain simplifying assumptions. There are alternative ways of transitioning from the fitness function to an expression for evolutionary change, for example, focusing on population genetics or quantitative genetics (see [17–21]). We will use a quantitative genetic approach here because it can be presented concisely while illuminating some key simplifying assumptions made in evolutionary game theory. Some of these assumptions could in fact be relaxed: for example, there may be an environmental component to the trait value *x* [19], and finite populations can be considered [17,24] but equations of similar form can still be recovered.

Here we assume that:

(i) the population is very large (idealized as an infinite population), so that stochasticity in fitness outcomes averages out over the population, and we can handle the function for expected fitness as if it had a deterministic effect on selection;
(ii) selection is *δ*-weak selection in the terminology of Wild & Traulsen [12], where fitness may be strongly influenced by the trait under consideration, but variance in the trait value is small at any given time, so there are only minor differences in fitness. We can then accurately estimate fitness of a focal individual with trait value *x* using a first-order Taylor polynomial [41] about the current mean trait value in the population, x*:
  2.2 $$w(x,x^{*})\approx w(x^{*},x^{*})+{\frac{\partial w}{\partial x}|}_{x=x^{*}}(x-x^{*});$$
  note that if the fitness function is itself linear in *x* (so that its radius of curvature is infinite) the above Taylor polynomial is exact and not an approximation; more generally, the larger the local radius of curvature of the fitness function (when compared to the variance in *x*), the more accurate the approximation (for the same reason we will later find that path coefficients and causal derivatives coincide in linear models); and
(iii) the trait value is passed on faithfully from parent to offspring (i.e. heritability equals one). This assumption overlaps with the phenotypic gambit [42].

We can then derive an expression for evolutionary change over a generation using the first covariance term of the Price equation [43]:

2.3 $$\Delta x^{*}=\frac{1}{\bar{w}}\mathrm{cov}(w(x,x^{*}),x)\approx \frac{1}{\bar{w}}\mathrm{cov}\left(\left[w(x^{*},x^{*})+{\frac{\partial w}{\partial x}|}_{x=x^{*}}(x-x^{*})\right],x\right)=\frac{\mathrm{var}(x)}{\bar{w}}{\frac{\partial w}{\partial x}|}_{x=x^{*}},$$

where we have used the observations that x*, *w*(x*, x*) and ∂*w*/∂*x*|*_x_*_=__x__*_ are constants over the population (i.e. every individual experiences the same population mean value x*, so that covariance with x* and with functions of x* over the population must equal zero) and that cov(*x*, *ax*) = *a* var(*x*) where *a* is a constant. We could additionally use the approximation $\bar{w}\approx w(x^{*},x^{*})$ when variance is small (see also [19–21]), but in our analysis this is not necessary.

An equation of generally similar form arises from population genetic and quantitative genetic considerations, and from the ‘adaptive dynamics’ framework [15,17,21]. We can see that because $\mathrm{var}(x)/\bar{w}$ is always non-negative, the derivative alone determines the direction of evolutionary change. Using the symbol ∝ for proportionality, we can write

2.4 $$\Delta x^{*}\propto {\frac{\partial w}{\partial x}|}_{x=x^{*}}.$$

The equations in this section, therefore, tell us that under typical assumptions of EGT, variation in fitness and the evolutionary response to selection are both proportional to the derivative $\partial w/\partial x{|}_{x=x^{*}}$. This expression of evolutionary change tells us how we can expect a trait value to evolve, given a hypothesis on how it is related to fitness, encapsulated by the fitness function *w*. When the derivative in equation (2.4) takes on a positive value, the trait value increases and vice versa. When the derivative equals zero, we have a candidate for an evolutionary endpoint. This does not guarantee stability of these endpoints [3,11,21,44], but in this article, we leave stability considerations aside and focus on fitness, the expression for evolutionary change (equation (2.3)), and their relation to concepts in causal modelling theory.

We note that when the population is composed of different classes (e.g. age classes, sexes, castes such as workers, queens, etc), instead of a simple fitness function we must use a weighted average over the different classes, where the weights are reproductive values: this accounts for potential differences in the long-term genetic contributions of different classes while retaining focus on an expression of one-generation evolutionary change (e.g. [11,17,22,45]). Although not the focus of our article, the concepts of class structure and reproductive value will briefly appear in the example on sex ratio evolution.

## 3. Path analysis, causal graphs, structural equation modelling and the causal derivative

A common interpretation of the derivative ∂w/∂x is that it represents how strongly the focal trait *x* causally affects fitness. This, however, is not necessarily the case in general. For example, assume phenotype *x* (say, weight) is influenced by rainfall *u* so that *x* = *u*, and fitness is also influenced by rainfall so that *w* = *u*^2^, but there is no direct influence from *x* to *w*. We could then write a valid equation stating *w* = *u*^2^ = *x*^2^ for this system, and differentiation gives d*w*/d*x* = 2*x*. But clearly this derivative says nothing about the causal relationship between the phenotype and fitness, and instead fitness and phenotype have a common cause and are said to be *confounded*. Even in cases where the phenotype does affect fitness, the derivative alone does not tell us how: it may be direct or indirect, or mediated via multiple pathways. Specifying, incorporating, and explicitly analysing the causal underpinning leads to a better understanding of how selection in a game theoretic model works.

Causal relationships are represented by path diagrams, where an arrow from one variable to another means that the former is a direct cause (often called a ‘parent’) of the latter. A sequence of arrows aligned in the same direction (such that *x*_1_ → … → *x_n_*) is called a *directed path*. When there is a directed path from *x* to *y*, *x* is a (possibly remote) cause of *y*. We limit our attention to acyclic diagrams, which have no directed path that ‘comes back’ to the same variable (so no variable is a cause of itself). There may be multiple directed paths between two variables. Indeed, in many game theoretic situations a focal trait can affect fitness through multiple pathways. For instance offspring sex ratio affects fitness via female and male offspring. Selection along these paths may act concordantly or discordantly, and in the latter case overall selection may even be zero if the causal effects via each pathway cancel each other out. One of the central motivations of this paper is to spell out the causal assumptions of game theoretical models in parallel both in graphical and mathematical terms, and to decompose the total fitness and selective effect of a focal trait into the path-specific effect of each of its paths.

The quantitative nature of each cause–effect relationship is modelled by a *structural equation* that determines the value of the effect variable from those of its parents, such that *x_i_* = *f_i_* (PA(*x_i_*), *u_i_*), where PA(*x_i_*) is the set of all parents of *x_i_* and *u_i_* is an unmodelled error term. Since in this paper, we are interested in explicating causal assumptions of *a priori* theoretical models rather than empirical hypotheses about actual systems, we assume that all relevant variables are modelled and error terms are independent from each other.

Once the causal diagram and its structural equations are specified, one may ask how a change or intervention in a particular variable affects others through paths connecting them. When all structural equations are linear, the path-specific effect can be calculated by Sewall Wright's [39,40] *method of path coefficients*. Suppose there is a directed path *x*_1_ → … → *x_n_*, and each causal link is linear so that $x_{i+1}=b_{i}x_{i}+{\;f}_{i+1}^{\prime }{\left(\mathrm{PA}(x_{i+1})\setminus x_{i},u_{i+1}\right)}_{\;}(1\;\leq i<n)$ where PA(*x_i+*1*_*)\*x_i_* is the set of parents of *x_i+*1*_* other than *x_i_* and ${\;f}_{i+1}^{\prime }$ is a (possibly nonlinear) function*_._* Then the path-specific effect *b* of this path is given by multiplying all the linear coefficients along the path, i.e. $b\;=\prod_{i=1}^{n-1}b_{i}$ [39,40]. This means that *x_n_* changes by *b* with a unit increment of *x*_1_ when every other path connecting them is held fixed. The total effect of *x*_1_ on *x_n_* is then given by summing up path specific effects over all the paths from *x*_1_ to *x_n_*.

This method was recently extended to nonlinear cases by Henshaw *et al*. [35] (see also [46]) in order to calculate the linear change in the effect variable induced by a small change in the cause variable. Let us again consider the path *x*_1_ → … → *x_n_*, but this time we allow the functional form of the links constituting the path to be nonlinear (but differentiable). Then the path-specific causal derivative of *x_n_* on *x*_1_ through this path, which is the change in *x_n_* owing to a small change in *x*_1_ when all other variables outside the path are held fixed, is given by $\prod_{i=1}^{n-1}\partial x_{i+1}/\partial x_{i}$. Note that this is an application of the chain rule [35,47] along the path. When *x_i_*_+1_ is a nonlinear function of *x_i_*, its derivative ∂*x_i_*_+1_/∂*x_i_* depends on the value of *x_i_*, so the causal derivative is a function of the variables constituting the path; while in the linear case, it is constant and reduces to Wright's method of path coefficients.

When there are multiple paths linking a cause *x* to an effect *w*, the total causal derivative of *w* with respect to *x* is given by summing up all the path-specific derivatives. Let π(*x*, *w*) be the set of all directed paths from *x* to *w*, and for each path *P* ∈ π(*x*, *w*) denote the variables along the path as (*x* = *p*_0_, …, *p_m_*_(*P*)_ = *w*) where *m*(*P*) is the length of the path. Then the total causal derivative is

3.1 $$\frac{\delta w}{\delta x}=\underset{P\in \pi (x,w)}{\sum }\overset{m(P)-1}{\underset{i=0}{\prod }}\frac{\partial p_{i+1}}{\partial p_{i}},$$

where the notation using the symbol *δ* indicates a special kind of partial derivative, where only non-descendants of *x* (i.e. those with no directed path from *x*) are held fixed [35]. The summation holds even if there are nonlinear interactions within each path or among paths in π(*x*, *w*).

The difference of the causal derivative from the standard one is that the former measures only the change in the target variable owing to a slight intervention on its causes. In the context of the study of adaptive evolution where the variables in question are fitness *w* (putative effect) and a trait *x* (putative cause), this means the causal derivative measures only selection *for x* [48] and ignores all other side effects arising from selection on correlated traits. This is not the case with the standard derivative. For instance, if the true causal structure is *x* ← *u* → *w* for some (possibly unobserved) confounding factor *u*, then *w* covaries with *x*, and thus the derivative dw/dx is usually non-zero, as we noted above. But the causal derivative δw/δx is zero everywhere, for there is no directed path from *x* to *w*. This reflects the fact that one cannot change the fitness value by intervening on *x* in this case (for their functional relationship is spurious). We will return to this topic at the end of this article.

## 4. A heuristic causal modelling scheme for evolutionary game theory

Now back to evolutionary game theory, where our interest is how, in a theoretical model, the fitness *w* of a focal individual changes in accordance with a slight change in a focal trait *x* when selection is frequency-dependent. Recall that frequency-dependence means that the fitness of a focal individual is determined not just by its own traits but also by those of others [11]. More specifically, in the types of models we explore, the selective pressure on the focal trait *x* is regulated by the population mean or resident quantity x*. A minimal and abstract causal model template for game theoretic setups then can be thought to include:

(i) the focal trait *x* and the population/resident trait x*, both of which may affect fitness; and
(ii) one or more variables that mediate the fitness contribution of the focal trait *x*. These ‘selective mediators’ regulate how *x* and x* affect fitness through nonlinear interactions. In addition, these mediator variables are themselves determined from *x* and x*.

**Figure 1.**
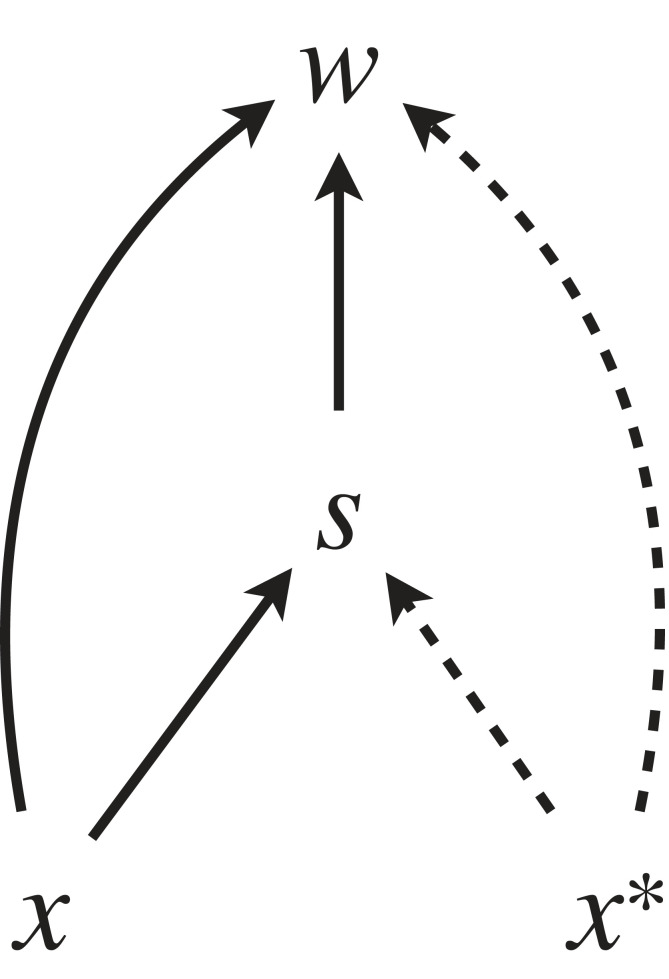
A heuristic model template that we use to analyse EGT models in the article. In frequency-dependent selection, the fitness effect of *x* is typically regulated by a selective mediator *s*, which in turn is determined by both the focal trait *x* and the resident quantity x* (which may or may not affect fitness by itself). Each solid edge (arrow) in the graph corresponds to a component of the causal derivative of *x*. The fitness function is commonly nonlinear in both *x* and x*. The selective mediator *s* may consist of multiple variables, and correspondingly there may be multiple pathways from *x* to *w*. Spelling out such mechanisms is the first task of building a causal model for EGT. Note that if there were no pathways (direct or indirect) from x* to *w*, the model would collapse to a simple optimization model (in the meaning of [33]). When we evaluate the game-theoretical derivative of equation (2.4), as well as the causal derivative of equation (3.1) with respect to *x*, only the solid paths appear explicitly as partial derivatives in the expression. In game theoretical terminology, we evaluate selection on *x* in an environment determined by the resident trait value. In causal modelling terminology, only the descendants of *x* are included in a causal derivative of *w* with respect to *x*.

Figure 1 is a simplified scheme of such causal models for EGT. This captures an abstract, minimal model of frequency-dependent selection, and is not meant to model any actual system. It should rather be considered as a heuristic template, illustrating in an abstract manner the general features shared by causal graphs that typically describe concrete models, as we will see in examples that follow. In particular, the selective mediators may take various forms which may be abstract, concrete, biotic, abiotic and so on. They may represent the combined outcome of the entries of a pay-off matrix (which may in turn encapsulate outcomes of contests), survival probability, fertilization rates, a ‘niche’, etc. There may also be multiple factors/variables that act as mediators, in which case there may be more than two directed paths and terms in the causal derivative. The mediators may be sequential or parallel, where the former corresponds to multiplication (∏) and the latter to the sum (∑) in equation (3.1). The first task of causal modelling is to identify relevant causal mechanisms and express them in terms of a causal diagram and structural equations.

Once the causal model is specified, one can calculate the causal derivative of fitness *w* with respect to the focal trait *x* as the sum of their respective path-specific derivatives; in this simple case δw/δx=∂w/∂x+∂s/∂x ∂w/∂s. To match the expression of evolutionary change (equation (2.4)) the path-specific derivatives are further evaluated at the population value $x=x^{*}$, and candidate equilibria can be found by solving the value of x* where the resulting expression vanishes.

Note that the causal derivative in evolutionary biology is typically used on relative fitness [35], but in this article, we will not be concerned with the distinction between relative and absolute fitness. We are not studying absolute magnitudes of path coefficients, but instead we are interested in their biological meaning, the insight we can gain from them on the causes of fitness and selection, and their relative magnitudes. For these purposes we can work with either relative or absolute fitness.

## 5. A causal model of a simple game

Let us illustrate the above generalized heuristic model structure in a simple two-player matrix game (which could, for example, represent the Hawk–Dove game [3] if appropriate pay-offs are chosen). Although explicit causal modelling and differential calculus do not bring new insight to this well-known example, they serve to illustrate how causal underpinnings of even very simple games with discrete strategies can be interpreted in line with the above heuristic template. Assume that in the two-player game the pay-off matrix is given as follows:

**Table:** 

	H	D
H	*a*	*b*
D	*c*	*d*

where each cell indicates the fitness pay-off (changes of fitness arising from the encounter) to an individual adopting the strategy indicated on the left, upon encounter with an opponent with the strategy above (e.g. Hawk or Dove: [3]). We can transform this matrix game into a continuous game by letting *x* be the probability of a mutant individual playing H, while x* is the same probability for the residents. Then the expected fitness of the mutant is

5.1 $$w(x,x^{*})=w_{0}+axx^{*}+bx(1-x^{*})+c(1-x)x^{*}+d(1-x)(1-x^{*}),$$

where $w_{0}$ is baseline fitness. This can be rearranged as:

5.2 $$\begin{array}{rl} w(x,x^{*}) & =w_{0}+x(ax^{*}+b(1-x^{*})-cx^{*}-d(1-x^{*}))\\ & +x^{*}(c-d)+d=xS(x^{*})+T(x^{*}), \end{array}$$

where $S(x^{*})=ax^{*}+b(1-x^{*})-cx^{*}-d(1-x^{*})$ and $T(x^{*})=$ $x^{*}(c-d)+d+w_{0}$.

**Figure 2.**
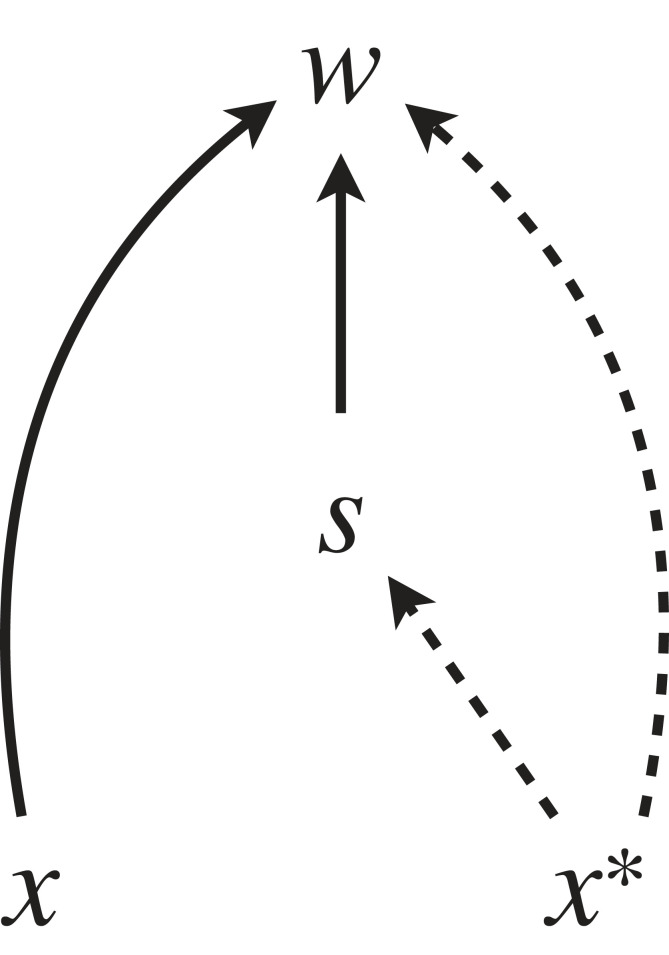
A causal diagram of the simple two-player (e.g. Hawk–Dove) game. *x* is the probability of a mutant individual playing Hawk, while x* is the same probability for the residents.

In the light of the causal diagram in figure 1, the resulting equation can be interpreted as a structural equation that shows that the resident value x*, given the pay-off matrix, affects fitness of the focal individual in two ways: one as an intermediate selective factor *S* acting multiplicatively with *x*, and second as an independent ‘base rate’ (denoted by *T*(x*)). It also tells that *S* does not depend on *x* in this particular example, so there is no arrow from *x* to *S* (figure 2).

Assuming that the directed path from *x* to *w* is not confounded, the causal derivative in this example coincides with the standard derivative:

5.3 $$\frac{\delta w}{\delta x}=\frac{\partial w}{\partial x}=ax^{*}+b(1-x^{*})-cx^{*}-d(1-x^{*})=S(x^{*}).$$

Equation (5.3) yields the direction of evolutionary change, and a necessary condition for the strategy x* to be an evolutionarily stable strategy is that this equation equals zero (note that usually we would need to additionally evaluate the derivative at $x=x^{*}$, but since *x* does not appear in equation (5.3), this would not change anything), with the solution

5.4 $$x^{*}=\frac{b-d}{b+c-a-d}.$$

This is just a reproduction of the classical result [3], but our presentation places it in the context of the general causal scheme of figure 1. The pay-off matrix can be seen as a kind of ‘environment’ that, combined with the resident value, determines selection on the focal trait. In EGT, this factor arises in the derivative of fitness with respect to the focal trait evaluated at the resident value. In the above simple example, the factor *S* was exactly equal to the derivative, since fitness is multiplication of *x* and *S*. Hence, causal analysis of this example using the causal derivative is not necessary to expose the causal structure, but it helps us by revealing a unified structure underlying a wide range of models. In general, the fitness function and the relevant causal factors may be much more complex, nonlinear, composed of multiple pathways and some selective mediators may be given only in abstract form with some general properties, rather than explicit functional form. In such cases, decomposing the complex causal nexus underlying the nonlinear fitness structure yields insight into how selection acts on the focal trait.

## 6. Exposing the logic of sex ratio models

The evolution of the sex ratio is a classic example of a problem that is game theoretical in nature, despite having been solved prior to the formal definition of EGT. The explanation for the prevalence and evolution of a 1 : 1 sex ratio is often attributed to Düsing [49] and Fisher [4], although several researchers played a role in the early decades of sex allocation theory [50] (see also [51] for a historical overview of Düsing's work, and [7] for a general exposition of sex ratio theory). Later (and already using some game-theoretic language) Hamilton [5] showed how female-biased sex ratios can evolve in a population where mating takes place in small, local groups before dispersal. Hamilton's work, however, led to a protracted uncertainty about the underlying causal explanation for the evolution of skewed sex ratios in his model, and this debate remained unresolved for years until a publication by Taylor [37] clarified the issue. Although not presented using the language of explicit causal modelling, the core of the paper amounts to an analysis of the causal derivative and an interpretation of partial derivatives it is composed of.

Here we will explicitly interpret Taylor's model using causal graphs and the causal derivative concept. While this does not change Taylor's results, it grounds it in formal theory of causal modelling, from the path coefficients presented by Wright [39] more than a century ago, to the recent causal derivative concept of Henshaw *et al*. [35]. With *x* indicating the evolving sex ratio, Taylor begins with a model of fitness of a mother producing *n*_d_ daughters and *n*_s_ sons:

6.1 $$w(x,x^{*})=n_{d}(x)D(n_{d}^{*}(x^{*}),n_{s}^{*}(x^{*}),n_{d}(x),n_{s}(x))+n_{s}(x)S(n_{d}^{*}(x^{*}),n_{s}^{*}(x^{*}),n_{d}(x),n_{s}(x)).$$

**Figure 3.**
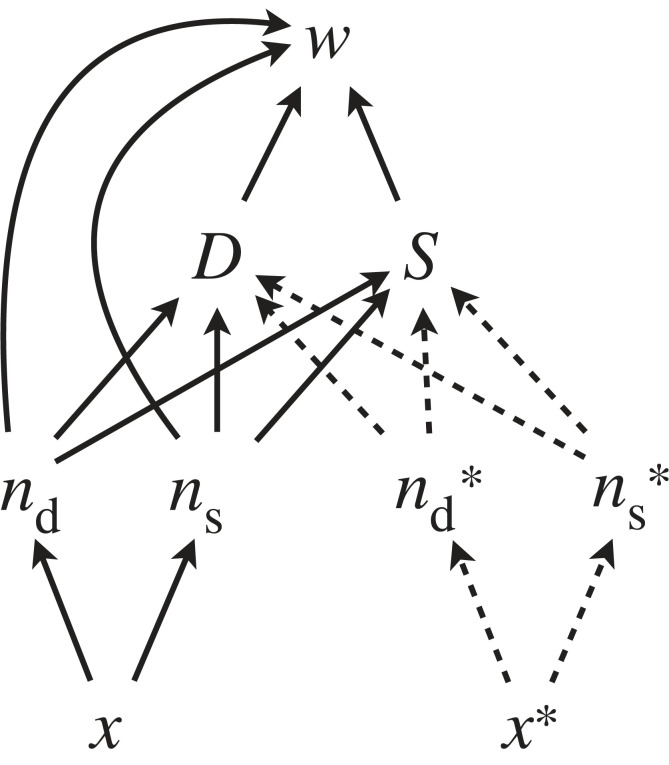
Taylor's sex ratio model. The expected genetic contribution *D* and *S* determines how daughters and sons contribute to fitness, and at the same time are determined by the numbers of daughters and sons of the focal and of resident individuals ($n_{d},n_{s},n_{d}^{*},n_{s}^{*}$). There are in total six directed paths from *x* to *w*, denoted by solid lines.

Here, *D* and *S* represent ‘selective mediators’ comparable to *S* in figure 1. These selective mediators determine how the number of daughters *n*_d_, and sons *n*_s_, contribute to fitness. In Taylor's model, these mediators are the ‘expected ultimate genetic contribution’ per daughter and per son, which are effectively the individual reproductive values of newly conceived individuals belonging to the female or male classes [45,52]. *D* and *S* are affected by the number of daughters and sons produced by resident individuals, as well as potentially by those produced by the focal individual. Finally, the number of daughters and sons are determined by the focal trait *x*, which is the proportion of reproductive resources allocated by the mother to the production of sons (equivalent to *r* in Taylor's notation), so that *n*_d_ is proportional to 1-*x* and *n*_s_ is proportional to *x*. These causal assumptions can be summarized by the causal graph in figure 3.

With this set-up, we are interested in the nature of the causal pathways driving x* towards an equilibrium value. In the causal graph of the sex ratio model (figure 3) there are six solid paths, composed of 10 edges. Correspondingly, the game-theoretical causal derivative consists of six terms, which are composed of 10 partial derivatives combined in various ways. Each of these 10 partial derivatives corresponds to a path coefficient in the graph above. They are $\partial n_{d}/\partial x,\;\partial n_{s}/\partial x,\;\partial D/\partial n_{d},\partial D/\partial n_{s},\partial S/\partial n_{d},\partial S/\partial n_{s},$ $D,S,n_{d}$ and $n_{s}$. Note that while the last four do not immediately look like partial derivatives, they are actually $\partial w/\partial n_{d}$, $\partial w/\partial n_{s}$, ∂w/∂D and ∂w/∂S respectively from equation (6.1).

The full game-theoretical causal derivative is

6.2 $${\frac{\delta w}{\delta x}|}_{x=x^{*}}={\left(\frac{\partial n_{d}}{\partial x}D+\frac{\partial n_{s}}{\partial x}S+\frac{\partial n_{d}}{\partial x}\frac{\partial D}{\partial n_{d}}n_{d}+\frac{\partial n_{s}}{\partial x}\frac{\partial D}{\partial n_{s}}n_{d}+\frac{\partial n_{d}}{\partial x}\frac{\partial S}{\partial n_{d}}n_{s}+\frac{\partial n_{s}}{\partial x}\frac{\partial S}{\partial n_{s}}n_{s}\right)|}_{x=x^{*}}$$

An omission in the original publication has been corrected here where the factors *n*_d_ and *n*_s_ were missing in the last four terms (but this does not affect the results of the original paper). The causal modelling approach does not change the conclusions of Taylor's paper, but we now have a formal link to path coefficients and other concepts of causal modelling. All these aspects together are helpful in reasoning about the causal structure of the sex ratio problem. The causal derivative decomposes the causal factors affecting fitness into several additive components, which are further composed of multiplicative components (path coefficients), and this kind of structure is considerably easier to read and understand as an additive causal model than a nonlinear fitness function. These various components are not just causal factors influencing fitness, but also causal factors influencing selection and evolutionary change which is also proportional to the causal derivative under the assumptions of EGT.

Taylor [37] gave the various partial derivatives appearing in equation (6.2) a causal interpretation (though not using formal language of causal modelling), and greatly clarified our understanding of the drivers of sex ratio evolution. The central insight was that the first two terms of equation (6.2) correspond to the classic, panmictic ‘Fisherian’ model, and that it is the last four terms that may cause deviations from 1 : 1 sex ratios. In the panmictic model the last four terms are all zero, while in Hamilton's [5] local mate competition model the last two deviate from zero, and cause female-biased sex ratios. In general, any of the last four terms in the equation can differ from zero if the focal mother's number of daughters or sons influences the long-term genetic contribution of her own daughters or sons. For these details, we refer readers to the original publication [37]. What our analysis adds to this is a clear, formal correspondence to causal derivatives, and hence path coefficients in a causal graph. These together can then be used to categorize and understand sex ratio models in a manner that would be far less intuitive using a nonlinear fitness function alone, and the combination of a game theoretical and causal modelling approach makes the analysis more broadly accessible.

We arrive at the same equation (6.2) by using the causal derivative formula (equation (3.1)), or by deriving the game theoretical derivative (equation (2.4)) for the sex ratio model. This does not, however, imply that the causal derivative is ‘just’ differentiation: instead, it is an equivalent of differentiation in a causal modelling context, whose components formally correspond to the path coefficients of Wright [39], and to the causal graph visually depicting the process (figure 2), and it can alternatively be defined as a limit using Pearl's [36] do-calculus (see the electronic supplementary materials for [35]).

## 7. Comparing the relative strengths of two causal pathways: sperm competition versus sperm limitation, and path-specific causal derivatives

Many long-standing and central game-theoretical questions relate to the causes and consequences of the evolution of gametic traits, such as their size and number [53,54]. For example, the evolutionary divergence of female and male gamete sizes (i.e. the evolution of anisogamy) has inspired mathematical models since the 1930s [55], many of them game-theoretical in nature [3,8]. These models gave rise to a recent debate on the relative importance of two components of selection in gamete evolution: gamete limitation and gamete competition. Gamete limitation refers to selection for improved fertilization success of gametes, while gamete competition refers to selection for increased share of fertilizations without necessarily increasing total fertilization success (e.g. [56]). Using anthropomorphic terminology, selection via gamete limitation has a cooperative streak to it while gamete competition is a more selfish selective agent [57], and the debate between these two aspects of selection reflects to some extent the debate between group selection and individual selection in evolutionary theory [58]. However, commonly models include only one or the other of these two aspects of selection, making it difficult to draw conclusions about their relative contributions to gamete evolution. When combined in a single model, both can drive selection in the same direction, selecting for increased numbers of gametes [59], but by itself this says nothing about their relative strengths.

The nature of these models is game-theoretical because when a mutant producing a deviant gamete size or number appears in a resident population, it interacts and competes with these resident individuals and their gametes. Thus causal derivatives in the game-theoretical context described above, and more specifically, path-specific causal derivatives [35] are a natural tool to compare the strengths of the two causal pathways. This kind of comparison has previously been done informally [60]. However, similar to Taylor's [37] sex ratio model, identifying model components with causal derivatives, and thus with path coefficients on a causal graph, gives the analysis a formal theoretical justification in a causal modelling context.

The basic model is set up so that male gametes (sperm) compete [61] for fertilizations of female gametes (eggs), an asymmetric perspective which is often justified when gametes are sufficiently diverged [62]. Though not shown here, a similar model can be applied in a symmetrical fashion, so that the mathematical form of equations does not differ for males and females [60].

**Figure 4.**
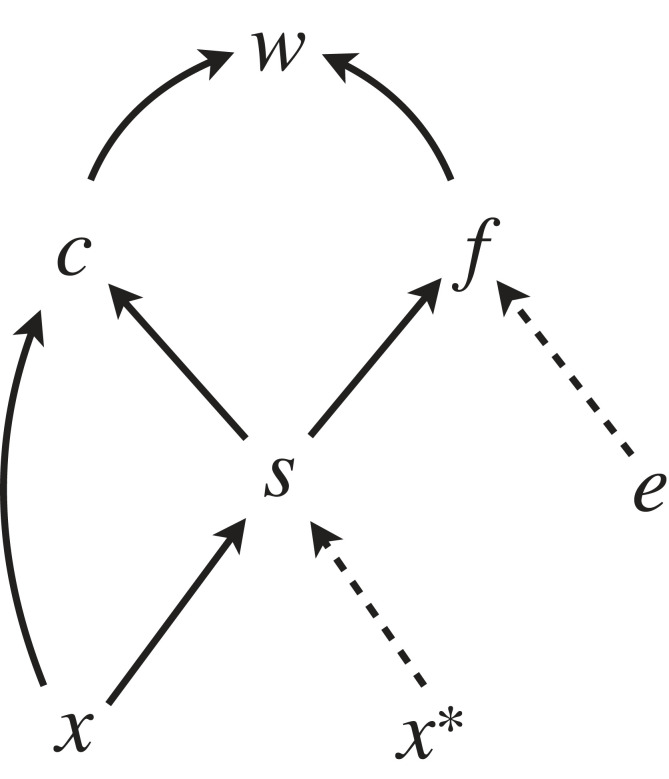
A causal model for gamete evolution. Sperm numbers of the focal and other individuals (x and x*) affect fitness through two distinct selection mechanisms, gamete limitation *f* and competition *c*, both of which are affected by the total sperm number *s*. The number of eggs the males are competing over is indicated by *e*.

Consider a rare mutant male competing for a set of *e* eggs with *N* − 1 resident males. This could be an external fertilizer with broadcast spawning that takes place in groups containing *N* males, or it could be an internal fertilizer with sperm storage, where each female receives *N* different ejaculates which mix such that each spermatozoon competes equally for fertilization of her *e* eggs. However, not all eggs are necessarily fertilized, and this fertilization success may depend on the total number or concentration of sperm *s*. Each resident male releases x* gametes, but a rare mutant instead releases *x* gametes. Our focal trait is *x*, whose fitness contribution is determined by the total number of eggs *e* as well as the total sperm number *s*, which in turn is a function of *x* and x*. We are interested in two factors mediating selection. The first is the total number of successful fertilizations *f*, which is a function of the total sperm number *s* and egg number *e*. The second mechanism through which the total sperm number affects the fertilization rate is sperm competition, denoted by *c*. The resulting causal diagram is shown in figure 4.

Given the causal diagram for this model (figure 4), our aim is to compare the relative contributions of paths passing via *c* to those passing via *f*. We proceed by assigning structural equations for each variable, based on reasonable biological assumptions. Since the competition occurs in a group of *N* males, of which *N* − 1 are residents, we have $s=x+(N-1)x^{*}$. To model *c*, the assumption that each sperm has an equal chance to fertilize a given egg implies ‘fair raffle’ sperm competition [61], so that $c(x,s)=x/s=x/(x+(N-1)x^{*})$. Of *f* we need only make a minimal assumption that it is a concave (decelerating or saturating) function of *s*, which is strongly implied by theoretical work spanning several decades (reviewed in [62]). For instance, a commonly used fertilization function is f(s,e)=e(1−exp(−as)), where *a* is a positive parameter. However, we do not need to assume this or any other particular form, beyond the requirement of concavity, to make progress in the analysis. Under these assumptions the fitness function becomes:

7.1 $$w(x,x^{*})=f(s,e)c(x,s)=f(s,e)\left(\frac{x}{s}\right),$$

where the resident trait value acts via the total sperm number $s=x+(N-1)x^{*}$. While the fitness function (7.1) might seem superficially relatively simple, it is in fact difficult to directly assess the relative effects of gamete limitation and gamete competition on fitness and selection: fitness is a product of two functions *c* and *f*, which are typically both nonlinear. However, we can again examine the causal derivative and its decomposition consisting of partial derivatives (path coefficients) for each edge in figure 4. We can then compare the sum of the paths from *x* to *w* passing via *c* to the single path from *x* to *w* passing via *f*, or in other words compare the path-specific causal derivative of *w* on *x* via paths that pass through *c* to those that pass through *f*. By definition, the path-specific causal derivative for a set of paths (say, *H*) tells us the rate at which fitness changes owing to changes in *x*, while holding all paths outside of *H* fixed [35]. The full causal derivative for this model is:

7.2 $${\frac{\delta w}{\delta x}|}_{x=x^{*}}={\left(\frac{\partial s}{\partial x}\frac{\partial f}{\partial s}c+\frac{\partial c}{\partial x}f+\frac{\partial s}{\partial x}\frac{\partial c}{\partial s}f\right)|}_{x=x^{*}},$$

and we can identify the first term in the brackets as that corresponding to the path passing via *f* in figure 4, while the sum of the last two terms corresponds to the paths passing through *c*. In other words, the two path-specific causal derivatives we are interested in are ∂s/∂x (∂f/∂s) c and ((∂c/∂x)f+∂s/∂x(∂c/∂s)f), evaluated at $x=x^{*}$. We haven't explicitly defined *f*, but surprisingly it can be shown that as long as *f* is a concave function of *s,* the sum of the last two terms exceeds the first term provided N≥2. In other words, as long as the focal individual faces competition from at least one other individual, selection via sperm competition tends to prevail over selection via sperm limitation. Details of the derivation are found in [60]. Again, the informal and intuitive justification of the original model is transferred to a more rigorous causal modelling context with concrete interpretations of model components as edges and paths in a causal graph and corresponding path coefficients (figure 4).

## 8. A causal modelling perspective on kin selection in continuous games

In the preceding sections we have assumed that when a rare mutant individual appears in the population, its fitness is influenced by the resident (or average) population strategy: fitness-affecting interactions take place with random population members of average phenotype x*. In other words, a rare mutant arising in the population has a negligibly low probability of interacting with another rare mutant (though its own gametes or offspring may interact with each other, as in the preceding examples). This assumption is broken if interactions are structured in a non-random manner so that rare mutants have an elevated probability of interacting with each other. For example, dispersal could be limited, so that related individuals tend to stay close to each other, or (extended) family members could otherwise non-randomly interact with each other [17,23,24,63]. In such cases, fitness of the focal individual is affected not only by its own and the resident phenotype, but potentially also by the phenotypes of its neighbours who may have an inflated likelihood of carrying the same mutation as the focal individual, resulting in correlated genotypes and phenotypes among interacting individuals. This is the idea behind kin selection [63] which is perhaps best known for providing an explanation for the evolution of altruistic behaviours, but which is now a central part of mathematical evolutionary theory and well-integrated with many other aspects of evolutionary theory in general ([23,24,34,64,65], to mention just a few from an enormous literature).

Kin selection theory has always had a game theoretical element to it, as both game theory and kin selection are built on the premise that the trait value of one individual can influence the fitness of another. Kin selection was nevertheless not well integrated with EGT until an influential paper by Taylor & Frank [22] showed how continuous game theory extends in a straightforward way to kin selection models. Taylor & Frank's method is an example of a ‘direct fitness’ [52] or ‘neighbour-modulated fitness' [63] approach to kin selection, as opposed to an ‘inclusive fitness’ [52,63] approach. Inclusive fitness is an actor-centred approach, which focuses on the effect that the trait of the focal individual (actor) may have on related individuals (recipients). Direct or neighbour-modulated fitness, in contrast, is a recipient-centric approach and focuses on the effect that the traits of related individuals (actors) may have on the fitness of the focal individual (the recipient) [22,24,52,64]. Direct fitness is the perspective on kin selection that integrates more seamlessly with EGT, and it will be our focus here.

**Figure 5.**
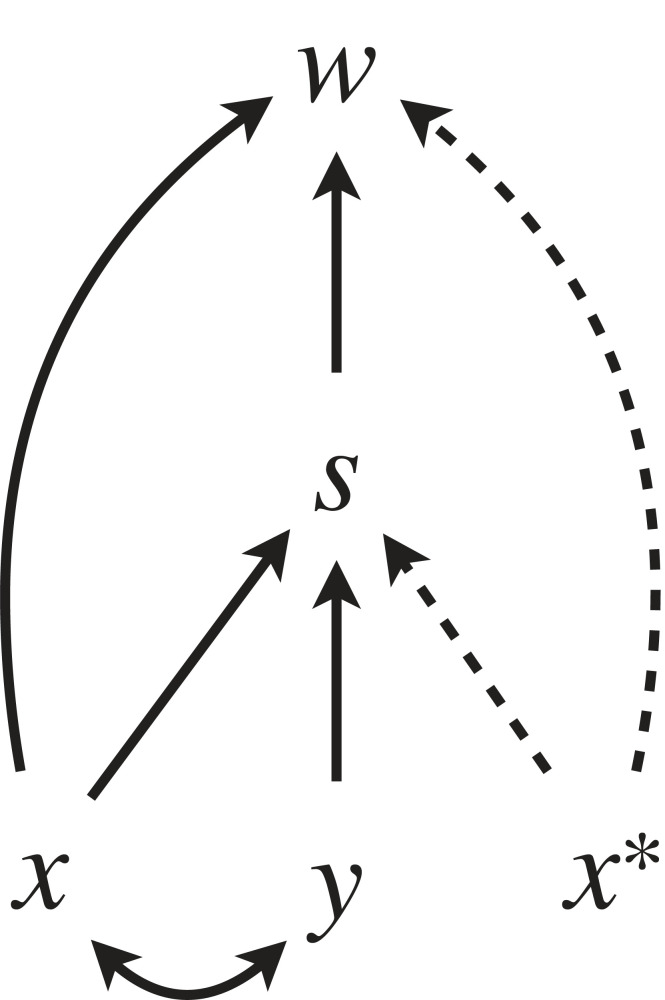
A causal model for kin selection, following the heuristic scheme of figure 1. The general model derivation in the main text does not explicitly include the intermediate mechanism *s,* but it is implicitly included in the total fitness effect of *x* and *y*.

In a direct fitness kin selection model, we focus on the fitness of a focal individual, similar to the usual game theoretical model. However, in addition to the fitness effect owing to the focal value *x* itself and owing to the resident trait value x*, we must account for the effect that neighbours with correlated phenotypes (*y*) may have on the fitness of the focal individual. This correlation is denoted by a double-headed arrow. The resulting causal model is given in figure 5.

To derive the selection differential in this set-up, one must consider two causal derivatives δw/δx and δw/δy each corresponding to fitness effects caused by *x* and by *y* (which may be correlated with *x*). In the absence of confounding, these correspond to the partial derivatives ∂w/∂x and ∂w/∂y of a first order multivariable Taylor polynomial [41] about the current mean trait value in the population $\left(x^{*}\right)$:

8.1 $$w(x,y,x^{*})\approx w(x^{*},x^{*}x^{*})+{\frac{\partial w}{\partial x}|}_{x=y=x^{*}}(x-x^{*})+{\frac{\partial w}{\partial y}|}_{x=y=x^{*}}(y-x^{*}).$$

Analogous to equation (2.4), the change in the mean trait value over a generation is

8.2 $$\begin{array}{rl} \Delta \bar{x}\approx & \frac{1}{\bar{w}}{\frac{\partial w}{\partial x}|}_{x=y=x^{*}}\mathrm{cov}(x,x)+\frac{1}{\bar{w}}{\frac{\partial w}{\partial y}|}_{x=y=x^{*}}\mathrm{cov}(y,x)\\ & =\frac{\mathrm{var}(x)}{\bar{w}}\left\{{\frac{\partial w}{\partial x}|}_{x=y=x^{*}}+{\frac{\partial w}{\partial y}|}_{x=y=x^{*}}\frac{\mathrm{cov}(y,x)}{\mathrm{var}(x)}\right\}\\ & =\frac{\mathrm{var}(x)}{\bar{w}}\{-c+br\}, \end{array}$$

where the last equality follows from noting that $\mathrm{cov}(y,x)/\mathrm{var}(x)=\beta_{yx}=r$ is a regression coefficient of relatedness [64,66,67], and denoting $\partial w/\partial x{|}_{x=y=x^{*}}=-c$ and $\partial w/\partial y{|}_{x=y=x^{*}}=b$. These are commonly called the ‘cost’ and ‘benefit’ terms of the famous ‘Hamilton's rule’, which states that there is positive selection for a trait if −c+br>0, and this can happen even if the trait is costly to its bearer (c>0) provided this cost is countered by a sufficiently high benefit (b>0) bestowed upon sufficiently close relatives (rb>c). These coefficients may be further decomposed into path-specific effects in line with the causal diagram in figure 5. Note the similarity of the equations (8.2) versus (2.3) as well as the causal graphs (figure 5 versus figure 1) of ‘standard’ game theory and kin selection in a game-theoretical framework. Again, if there are no causal pathways from *y* to *w* and x* to *w* (implying they also do not appear in the fitness function), the graph reduces to simple optimization (*sensu* [33]).

Taylor & Frank [22] showed how kin selection merges with game theory in this way, and made the application of the direct fitness method much easier than it had been previously. Their method also provided powerful tools for incorporating class-structure into kin selection models, thus offering a general recipe for analysing a range of complicated model scenarios. Interestingly, in their original publication, Taylor & Frank [22] seem to treat *y* as if it was actually a descendant of *x*: if we replace the double-headed arrow in figure 4 with a single-headed arrow pointing from *x* to *y*, we find that the causal derivative of *w* with respect to *x* collects all the effects included in the selection differential above, with the relatedness coefficient *r* replaced by the partial derivative ∂y/∂x. In terms of a causal graph, then, the substitution ∂y/∂x=r in Taylor & Frank [22] follows from calculating a causal derivative of *w* with respect to *x* on the causal graph such as that in figure 5 *as if x* is a cause of *y*. In practice, this computational trick leads to the same result. In a different paper Taylor [20] points out that the method of Taylor & Frank [22] treats *y* as if it is causally influenced by *x* which is not necessarily the case, and in general it only covaries with *x*.

## 9. Trans-generational kin selection games

The fitness effects related individuals can have on each other need not be confined to one generation. The fitness of an individual existing now can be influenced by the actions of another individual in the past via modifications of the environment. In fact, the possibility of modification of the environment is built into the very first causal graph of EGT we presented (figure 1, right side): the selective mediator *s* can represent an environmental factor which is potentially altered by the focal individual and/or the population at large via the resident value x*. This viewpoint is emphasized in particular by the ‘adaptive dynamics’ approach [15,68], where a separation of evolutionary and ecological timescales is one of the central assumptions [69] and allows the environment (as influenced by the resident population) to reach an equilibrium state over several generations and subsequently influence selection on the focal trait. This property of EGT is closely connected to the concept of niche construction. If we take a common, broad definition of niche construction as the modification of selective environments by organisms [70,71], niche construction is present even in the basic form of evolutionary game theory (figure 1) when *s* is considered as ‘the selective environment’. However, in this case there is little scope for selection to shape the environment in an adaptive fashion in the long term, or for ‘caring about the future’ in terms of altruism that extends across generations: after any alteration of the environment, selection again acts in a short-sighted and selfish fashion with no regard for the past or the future, often leading to outcomes where competition deteriorates the environment on which everyone depends (the 'tragedy of the commons': [72]).

A more interesting example of ‘niche construction’ can arise when kin selection acts across generations: individuals that exist at different points in time can still share genes and phenotypes owing to shared ancestry, permitting the evolution of trans-generational altruism [73] and the evolution of adaptive niche constructing traits [74], thus reducing the tragedy of the commons. Again, this can be considered an extension of game theory where the current environment is altered not only by those relatives living in the current generation (as in figure 5), but also by relatives who lived in past generations [73,74].

**Figure 6.**
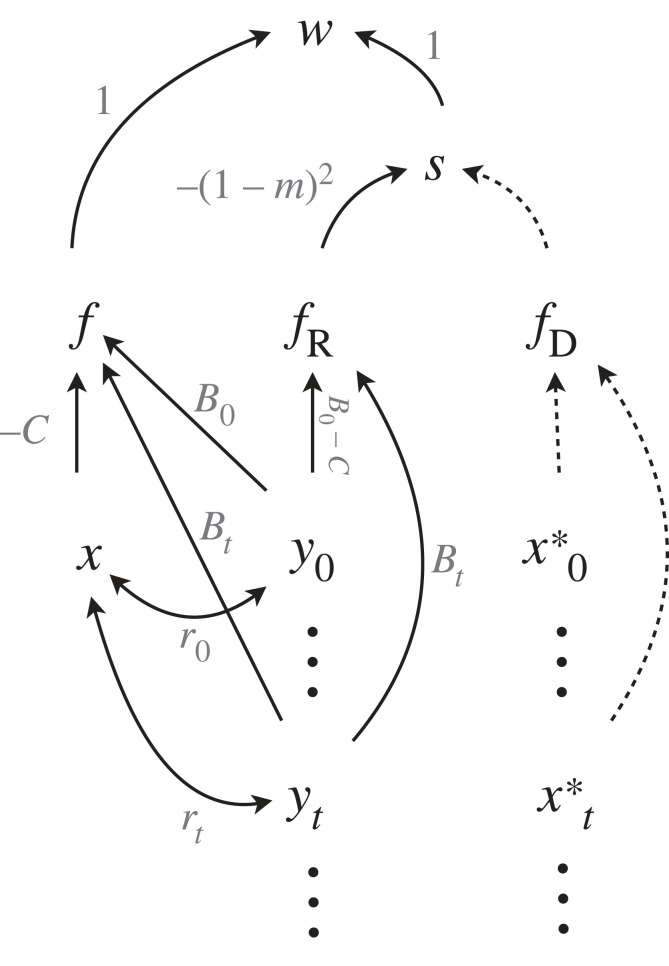
Trans-generational kin selection, where the fitness of the focal individual is influenced not only by contemporary relatives, but also by those who lived in previous generations and altered the environment. The relatedness between individuals in different generations can be computed as space–time relatedness coefficients [73,74] corresponding to each double-headed arrow in the graph. Our notation differs slightly from that of Lehmann [73]. Here, *x* is the phenotype of the focal individual, while *y_t_* is the average phenotype of individuals living in the focal deme *t* generations prior to the focal generation and *r_t_* is the relatedness between the focal individual and an individual living *t* generations prior to the focal generation in the same deme. x*_t_ is the population resident value *t* generations prior to the focal generation. *B_t_* is the fecundity benefit received from all actors expressing acts of helping in the focal deme *t* generations prior to the focal generation, while *-C* is the cost of an altruistic act. Note that the subscript 0 indicates ‘0 generations prior to the focal generation’, i.e. the focal generation itself. The probability of dispersal to another patch is indicated by *m*. Variables *f*, *f*_R_ and *f*_D_ are relative fecundities: that of the focal individual, the average relative fecundity of individuals in its deme, and the average ‘resident’ fecundity respectively. The competition for a breeding spot in a deme is indicated by *S*.

Figure 6 is a diagrammatic representation of a trans-generational kin selection model discussed in Lehmann [73]. In this model, individuals live in finite demes, from which juveniles disperse with probability *m*. Limited dispersal results in inflated relatedness between individuals in a deme, both within and between generations. The fecundity *f* of the focal individual with trait *x* is positively affected by the trait values *y*_0_ of individuals living in the same deme at the same time and $y_{t}$ of their ancestors at generation *t*. Variables $f_{R}$ and $f_{D}$ are average relative fecundities of individuals in the focal and non-focal (resident) demes respectively, which also have inputs from the past generations. In each deme, exactly *N* juveniles can reach adulthood so the population is held at a constant size, and this implies competition for breeding spots between juveniles. This competition is in turn influenced by the fecundities of individuals in the local deme, as well as ‘resident’ individuals immigrating from other demes, and we therefore denote the competition term as a ‘selective mediator’ indicated by *S*. In Lehmann's model, fitness is given by $w=f\;S(\;f_{R},f_{D})$, where *S* is a nonlinear function of $f_{R}$ and $f_{D}$. The focal individual is related to individuals in the same deme with the relatedness coefficient *r*_0_, and also to their ancestors in the *t*-th generation with *r_t_*.

With this model, the relevant causal derivatives are

9.1 $$\frac{\delta w}{\delta x}=\frac{\partial f}{\partial x}\frac{\partial w}{\partial f}\;,$$

9.2 $$\frac{\delta w}{\delta y_{t}}=\frac{\partial f}{\partial y_{t}}\frac{\partial w}{\partial f}+\frac{\partial f_{R}}{\partial y_{t}}\frac{\partial S}{\partial f_{R}}\frac{\partial w}{\partial S}$$

and the selection differential is proportional to

9.3 $$\frac{\delta w}{\delta x}+\;\frac{\delta w}{\delta y_{0}}r_{0}+\overset{n}{\underset{t=1}{\sum }}\frac{\delta w}{\delta y_{t}}r_{t},$$

where the first term is the direct cost for a focal individual, the second term contains the benefit from other individuals in the same generation as well as the negative effect of increased competition in the patch owing to overall increased fecundity, and the third term includes the accumulated benefits from prior generations as well as the effect of increased competition owing to the intergenerational fecundity benefit. If each partial derivative is evaluated according to figure 6 and accounting for the corresponding path coefficients, this reproduces Lehmann's [73] equation (3.1) which gives the direction of selection on a mutant allele. The main advantage of an explicit causal analysis for this model is the increased transparency it presents particularly for readers unfamiliar with the mathematical methods used in the study. In this model the transmission of benefits from the past is not explicitly described, but alterations of the model can make this transmission more explicit; for details on the space–time relatedness coefficients $r_{t}$, as well as model extensions we refer the reader to Lehmann [73,74].

As in the previous section, this is again a ‘direct fitness’ approach where we focus on the fitness of one recipient and collect the effects on their fitness of other individuals living in the same or past generations (figure 6). As before, we could take an ‘inclusive fitness approach’, where we focus on a single actor in the current generation and sum up its effects on fitnesses of relatives in the current and future generations. Both calculations result in the same evolutionary change [74].

## 10. Confounding, the causal derivative, the game-theoretical derivative and the phenotypic gambit

While to an evolutionary biologist it may seem almost obvious that the game-theoretical derivative $\partial w/\partial x{|}_{x=x^{*}}$ has an explicit causal meaning, it may be far from obvious to an expert in causal modelling (conversations between the authors of this article serve as evidence). Indeed, given the possibility of confounding discussed above, a causal theorist is entirely justified in their scepticism towards the causal meaning of a derivative. However, the evolutionary biologist may equally justifiably say that the whole point of a game theoretical model is to construct a causal hypothesis on the cause of fitness and analyse the consequences of this hypothesis. The focal trait is assumed *a priori* to have a fitness effect without confounding. The idea of a causal model (starting with the additive fitness pay-offs in a matrix model) is perhaps so deeply ingrained in EGT that it is rarely explicitly mentioned. Possibly for this very reason the causal basis of EGT is not usually given much attention in a formal sense (although exceptions exist: see Frank [23] and Queller [66] for examples in kin selection theory).

Both viewpoints are therefore valid. It is true that lack of confounding is almost implicit in evolutionary game theory, but it is nevertheless a substantive assumption about the causal structure of the problem. The fact that we can write *w* as a mathematical function of *x* does not imply a causal relationship between the two. In EGT we assume such a causal relationship does exist, and the fitness function or pay-off rule represents this causal relationship. Under this assumption, the identification of the game-theoretical derivative with the causal derivative is justified.

Put another way, none of this is a problem in the theoretical world which was built by the modeller, which may only exist on pen and paper, and where the modeller decides the rules, some less consciously than others. However, when we try to apply this model to an actual population, we do not know *a priori* that there is no confounding that may cause the real world to deviate from the theoretical world. It is at this intersection of the theory world and real world where the distinction between the standard derivative and causal derivative becomes crucial.

A more commonly discussed aspect of the theory–reality interface in EGT (and phenotypic models in general) is the so-called *phenotypic gambit* [42,75,76]. In essence, the phenotypic gambit is the research strategy of studying organismal evolution with little or no knowledge of the actual genetic architecture of the trait in question [75]. We assume we can get precise enough predictions and explanations by working with the phenotype alone. The assumptions inherent in the phenotypic gambit therefore relate to the genotype–phenotype map. By contrast, the problem of confounding concerns the causal relationship between the phenotype and fitness. These are distinct assumptions about the causal structure of a target population, and our point here is that the confoundedness may bias predictions of a game theoretic model in a way different from phenotypic gambit.

## 11. Conclusion

Our aim with this article has been to build a bridge between evolutionary game theory of continuous quantitative traits on one hand, and formal theory of causal modelling on the other. In this way, the causal structure and meaning of game theoretical models in evolutionary biology becomes more readily understandable to those unfamiliar with the mathematical side of the modelling method; conversely, our intention is also to bring the tools and concepts of causal modelling within reach of game theoreticians. Game theoretical models in evolutionary biology have been analysed from a causal perspective previously, either in description of general methodology (e.g. the use of path diagrams in kin selection models of [23,77]), or implicitly in clarifying the nature of debated issues in evolutionary theory and in picking apart the detailed biological mechanisms driving selection in a given model (e.g. the analysis of sex ratio evolution in [37]; interpretation of dispersal models in [38]; comparison of causes of selection in gamete evolution in [60]). However, such approaches have often been informal, and the justification for drawing causal conclusions from mathematical expressions in a given model may remain vague: partial derivatives and their combinations seem to have a causal meaning and yield biological insight, and their relative magnitudes can inform us about the relative contribution of different pathways to selection, but it is difficult to pin down exactly why this works.

We have shown that there is scope to unify evolutionary game theory and causal modelling in a more formal fashion, which clarifies the nature of modelling methods, facilitates the use of unified, common language across such analyses, and justifies the use of explicitly causal language in game theoretical research. The same concepts used in e.g. disentangling causal relationships from empirical data apply to the causal analysis of a game theoretical model. A causal diagram is often more readily understandable than a sum of the products of partial derivatives, while mathematical expressions carry more detailed information than a causal diagram. Their combination can be more than the sum of their parts. The use of causal graphs makes the structure of various types of game-theoretical models more readily understandable and approachable to non-specialists, from the basic set-up of figure 1 to the kin selection model of figure 5 and the intra-generational kin selection and niche construction model of figure 6. The causal approach thus helps us categorize modelling methods and understand their relationships to each other.

We have seen how the causal derivative recently introduced by Henshaw *et al*. [35] is typically implicit in game theoretical models: confounding effects are usually assumed to be absent, so that the game-theoretical derivative corresponds to the causal derivative. This in turn gives formal justification for the interpretation of partial derivatives in a game theoretical model as Wright's path coefficients [39,40] of the corresponding edges in a causal graph, while products of these path coefficients make up path-specific effects (path-specific causal derivatives: [35]). We can then recast, for example, Taylor's [37] exposition of the logic of sex allocation models as a visually intuitive path diagram where partial derivatives become path coefficients. Similarly, we can interpret sums of products of partial derivatives as path-specific effects passing through a particular node in a causal graph, as was done informally in a game-theoretical comparison of the drivers of gamete evolution [60].

Evolutionary game theory with its extensions into kin selection [22], class-structured populations [22,45], the evolution of niche-constructing traits [73,74] and with its ever-increasing scope [11] has become one of the foremost methods for illuminating the nature of adaptation and the appearance of ‘design’ in nature, linking a model of evolutionary change over one generation to a long-term view of adaptation [17]. One of its most attractive features is that it makes the analysis of almost arbitrarily complex nonlinear models with frequency dependence tractable. A causal analysis of the mathematical components (partial derivatives) of such a model can yield insight that can be next to impossible to gain otherwise [38]. Recent advances in the causal analysis of nonlinear interactions [35] fit organically into this framework and provide a formal bridge between methods of modern evolutionary game theory, and those of causal modelling.

## Data Availability

This article has no additional data.
